# Catalysis: transition-state molecular recognition?

**DOI:** 10.3762/bjoc.6.117

**Published:** 2010-11-03

**Authors:** Ian H Williams

**Affiliations:** 1 Department of Chemistry, University of Bath, Bath BA2 7AY, United Kingdom

**Keywords:** catalysis, computational simulation, enzymes, molecular recognition, transition state

## Abstract

The key to understanding the fundamental processes of catalysis is the transition state (TS): indeed, catalysis is a transition-state molecular recognition event. Practical objectives, such as the design of TS analogues as potential drugs, or the design of synthetic catalysts (including catalytic antibodies), require prior knowledge of the TS structure to be mimicked. Examples, both old and new, of computational modelling studies are discussed, which illustrate this fundamental concept. It is shown that reactant binding is intrinsically inhibitory, and that attempts to design catalysts that focus simply upon attractive interactions in a binding site may fail. Free-energy changes along the reaction coordinate for S_N_2 methyl transfer catalysed by the enzyme catechol-*O*-methyl transferase are described and compared with those for a model reaction in water, as computed by hybrid quantum-mechanical/molecular-mechanical molecular dynamics simulations. The case is discussed of molecular recognition in a xylanase enzyme that stabilises its sugar substrate in a (normally unfavourable) boat conformation and in which a single-atom mutation affects the free-energy of activation dramatically.

## Introduction

“Molecular recognition of transition states” was the title of a paper presented by Kirby [1] at a discussion held in April 1993 on the chemistry of biological molecular recognition; he addressed the fundamental question of how enzymes lower the free energies of the transition states for the reactions they catalyse, with reference to his own elegant experimental studies on catalysis. In March 1991, at a workshop held under the auspices of the Science and Engineering Research Council’s Molecular Recognition Initiative, I presented a paper on theoretical modelling of transition states for biochemical processes, which included a computational model for carbonyl reduction catalysed by lactate dehydrogenase [2]. The abstract for this workshop presentation began with the following sentence: The key to understanding of the fundamental processes of catalysis is the transition state; indeed, “catalysis is a transition-state molecular recognition event”. The present paper discusses cases of methyl transfer and of glycoside hydrolysis to illustrate and to update the same theme from a computational point of view.

## Discussion

**Figure 1:**
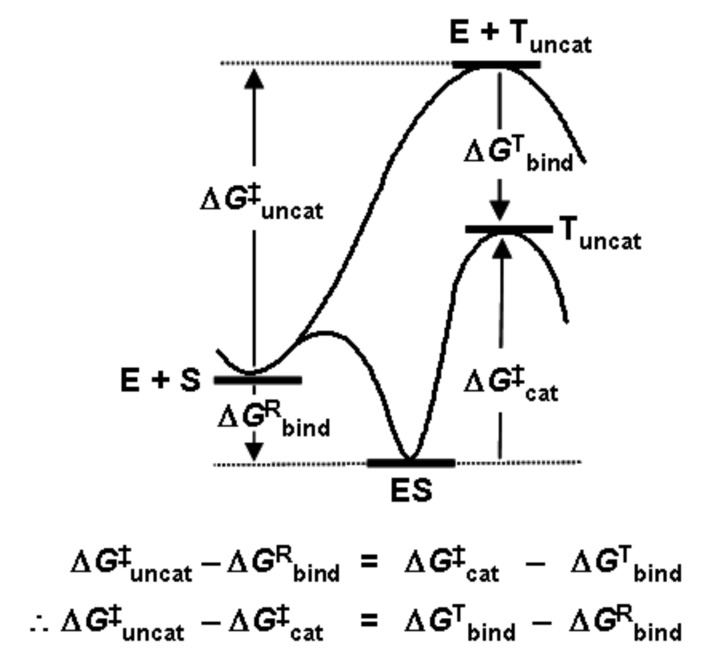
Free energy profiles for reactions of substrate S uncatalysed and catalysed by enzyme E, showing how the barrier height reduction is equal to the binding energy for transition state T offset by the binding energy for the reactant state R.

The transition state is of strategic importance within the field of chemical reactivity. Owing to its location in the region of the highest energy point on the most accessible route between reactants and products (Figure 1), it commands both the direction and the rate of chemical change. Questions of specificity and catalysis may be answered by knowledge of the structure and properties of the TS.

Computational chemistry provides techniques for the generation and exploration of the multi-dimensional energy surfaces that govern chemical reactivity; energy minima and saddle points can be located and characterised, and the pathways that interconnect them can be determined. A rigorous distinction should be drawn between a TS (corresponding to a bottleneck on a free energy surface) and a transition structure (corresponding to a saddle point on a potential energy surface). The commonly assumed identity between the two terms is often reasonable for small, “simple” systems in vacuum, for which it may be sufficient to model the TS by first finding a transition structure and then evaluating its molecular partition function by QM computations. However, it would be quite wrong to neglect the distinction for “complex” systems, for which the free energy of the TS may not be evaluated using simple analytical expressions for partition functions determined for a single transition structure. Enzyme catalysed reactions in solution are of this nature, and it is necessary to take averages over an extensive sampling of configurational space in order to obtain the changes in free energy that dictate their reactivity.

It was Linus Pauling who suggested that the catalytic activity of enzymes was due to structural complementarity with the TS rather than the reactant state of the substrate [3]: “enzymes are molecules that are complementary in structure to the activated complexes of the reactions they catalyse … [which] would thus lead to a decrease in its energy, and hence to a decrease in the energy of activation” [4]. A corollary to this insight was provided by W. P. (Bill) Jencks, who noted that a catalyst might be synthesised by raising an antibody to a hapten resembling the TS of the reaction to be catalysed: “the combining sites of such antibodies should be complementary to the TS and should cause an acceleration by forcing bound substrates to resemble the TS” [5]. However, the clear logical implications of the notion of TS complementarity for understanding the origins of enzyme catalytic power were described eloquently (but with a friendly tongue in cheek) by R. L. (Dick) Schowen as the “fundamentalist” position in contrast to the “canonical” view of Jencks and others. He asserted that “the entire and sole source of catalytic power is stabilisation of the TS” [6], which implied not only that reactant-state binding interactions were by nature inhibitory and only wasted catalytic power (Figure 1), but also that the particularities of any events occurring along paths between reactants and TS (termed as the “microhistory” of the reaction [7]) are irrelevant to the catalysis itself. Theories within the “canon” of enzyme catalysis tend to omit or at least de-emphasise the TS, focussing instead on some sort of reactive complex en route from reactants to TS. For example, Bruice’s “near-attack conformation” concept highlights a particular structure (a “NAC”) which behaves as a “turnstile through which the ground state must pass to enter the TS” [8]. One might consider, however, that this amounts to redefinition of the dividing surface between reactants and products as the NAC rather than the TS, but without providing any means for locating and characterising it. In my opinion, the TS is already well defined and continues to serve well as the focus of the present discussion.

Recently, some authors have sought to go “beyond the Pauling paradigm” by noting that “enzymes enter into reactions with substrates and do not merely complement the transition states of the uncatalysed reactions” [9]. The implication seems to be that the notion of TS complementarity and TS stabilisation as the source of enzyme catalytic power ignores any interactions between an enzyme and the substrate in the reactant state. However, careful reading of Pauling’s own words reveals that his views on enzymes follow a discussion of structural complementarity between an antibody and its antigen, and that his statement (quoted above) regarding complementarity between an enzyme and the “activated complex” of the catalysed reaction is in turn followed by this sentence [3]: “If the enzyme were completely complementary in structure to the substrate, then no other molecule would be expected to compete successfully with the substrate in combining with the enzyme, which in this respect would be similar in behaviour to antibodies; but an enzyme complementary to a strained substrate molecule would attract more strongly to itself a molecule resembling the strained substrate molecule than it would the substrate molecule.” This clearly implies a consideration of the relative extent of binding interactions of the reactant state and TS with an enzyme, and of the inhibitory nature of the former.

The essential importance of *preferential* TS stabilisation was absolutely explicit in Schowen’s treatment [6]: “A complete understanding of enzyme catalysis … resolves into a characterisation of two binding processes: that for the transition state, which yields a model for catalysis, and that for the reactant state, which yields a model for … inhibitory effects … The differential stabilisation of the transition state (total stabilisation of the transition state minus stabilisation of reactant species) always gives the catalytic acceleration.” Recently, Simón and Goodman [10] have astutely observed that an optimal catalyst does not simply maximise TS stabilisation per se, but rather achieves a maximal reduction in barrier height by means of differential stabilisation. The cases discussed below all exemplify TS molecular recognition and stabilisation *relative* to the reactant state.

### Catalyst design: preferential TS binding

**Scheme 1:**
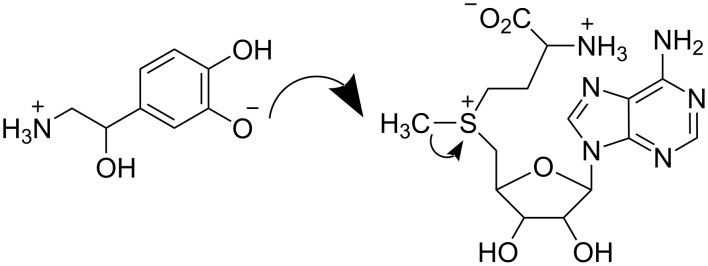
S_N_2 methyl transfer from SAM to catechol catalysed by COMT.

Methyl group transfer from an electrophile to a nucleophile by an S_N_2 mechanism is an archetypal reaction in organic chemistry and an important process in biochemistry. Catechol-*O*-methyl transferase (COMT) catalyses methyl transfer from *S*-adenosylmethionine (SAM) to a catechol (Scheme 1), and this reaction manifests an unusually large inverse secondary kinetic isotope effect as compared with a model, uncatalysed reaction in solution: the isotope effect *V*^CH3^ /*V*^CD3^ = 0.83 ± 0.05 for methylation of 3,4-dihydroxyacetophenone with SAM at 37 °C catalysed by COMT was found [11] to be more inverse than the value of *k*_CH3_ /*k*_CD3_ = 0.97 ± 0.02 for methylation of methoxide ion by *S*-methyldibenzothiophenium ion at 25 °C in methanol [12]. According to the orthodox view, Schowen and co-workers interpreted these observations in terms of a tighter S_N_2 transition state for the COMT-catalysed reaction than for the non-enzymic reaction, and consequently proposed the “compression hypothesis” for enzymic methyl transfer as a possible explanation [13].

**Figure 2:**
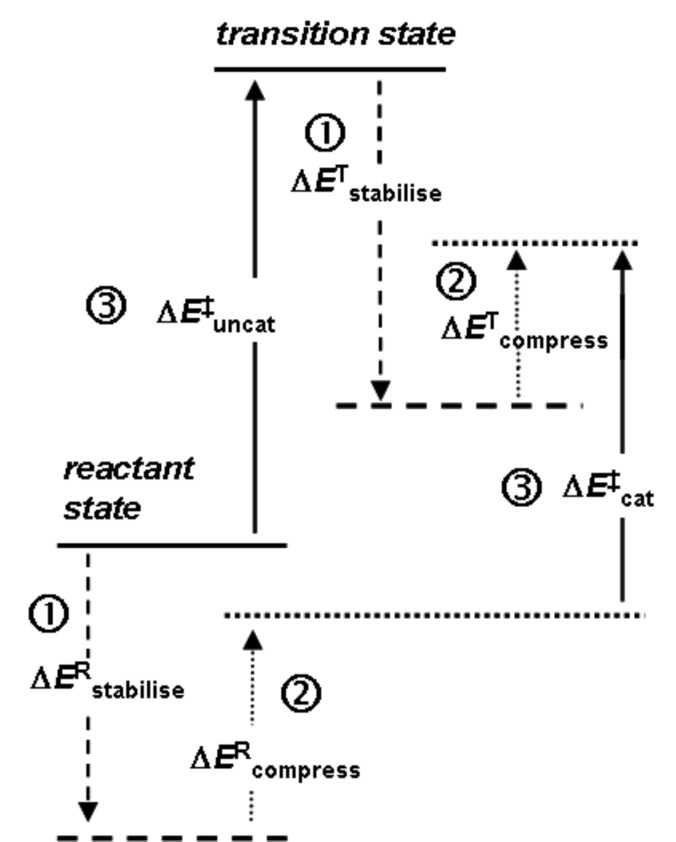
Energetic analysis of the compression hypothesis for enzyme-catalysed methyl transfer.

As outlined above, the power of any catalyst derives fundamentally from its ability to stabilise the TS relative to the reactant state, as compared with the uncatalysed reaction. This requires effective discrimination between the reactant state and the TS. In the case of methyl transfer, stabilising enzyme-substrate interactions (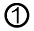 in Figure 2) probably do not provide any significant degree of discrimination, since the geometrical and electronic changes occurring do not provide sufficient differences; thus

[1]
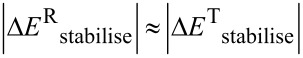


The key proposal of the compression hypothesis is the following: if the TS for S_N_2 methyl transfer is more plastic than the reactant state for the catalysed process, then mechanical compression by the enzyme (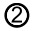 in Figure 2) might destabilise the reactants more than the TS. In other words, the energetic penalty for deforming the structure to a given degree is greater for the reactant state than for the TS:

[2]
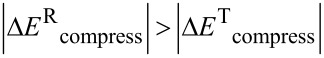


The net effect (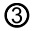 in Figure 2) is the reduction of the barrier for the catalysed reaction as compared with that for the uncatalysed process:

[3]
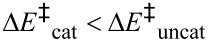


As a consequence of (intrinsically unfavourable) compression of the S_N_2 TS in the enzymic reaction, the enzyme is able to distinguish the TS structurally from the preceding reactant state and the succeeding product state in order to stabilise the TS specifically. Thus, compression may serve to achieve efficient catalysis, with a large *V*_max_ at the expense of a slight reduction in *V*_max_/*K*_m_. The importance for enzyme catalysis of destabilisation as well as binding has also been noted by Jencks [14].

**Figure 3:**
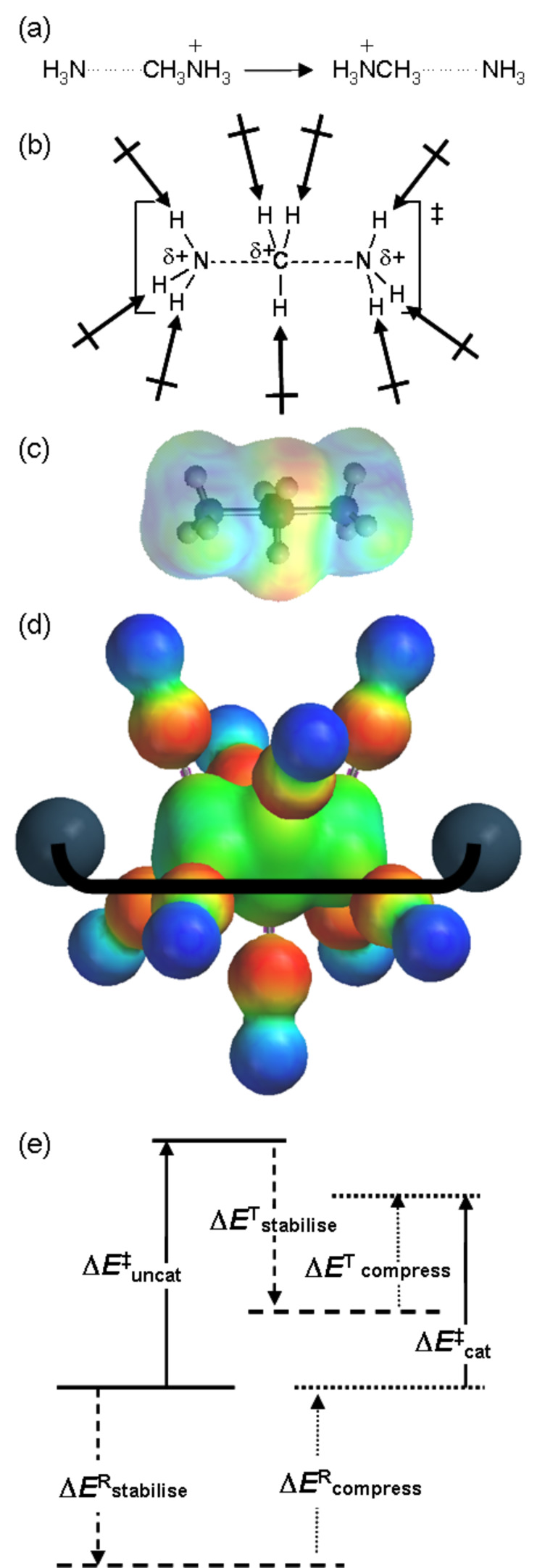
Catalyst design for methyl transfer: (a) the reaction to be catalysed; (b) dipoles favourably aligned with the transition structure; (c) electrostatic potential plotted on the isodensity contour surface of the transition structure; (d) electrostatic potential (on isodensity contour surface) of the complex of the transition structure within a frozen array of dipoles, together with a pair of inert-gas atoms; (e) preferential TS stabilisation as the net result of stabilising (attractive) and destabilising (repulsive, compressing) interactions.

Some years ago I performed an ab initio Hartree–Fock investigation [15], intended to test the validity of the compression hypothesis; this exercise amounted to the computational design of a catalyst for the identity S_N_2 methyl transfer from methylammonium to ammonia (Figure 3a). The transition structure for this reaction has an overall positive charge, and a reasonable strategy for its stabilisation seemed to be to construct an array of point charges, such that each N–H or C–H bond was perfectly aligned with the negative end of a dipole (Figure 3b). However, when both the transition structure and the ion-molecule reactant complex were reoptimised within the frozen array of point charges, it transpired that the stabilisation energy Δ*E*^R^_stabilise_ of the latter was greater than the stabilisation energy Δ*E*^T^_stabilise_ of the former (Figure 3e). Unintentionally, the barrier for S_N_2 methyl transfer with the “catalyst” was higher than that without: inhibition, or anti-catalysis, had been achieved. With hindsight, it may be seen from the electrostatic potential of the transition structure (represented by colour on an electron density contour in Figure 3c), that the transferring methyl group is unlikely to interact favourably with the dipoles intended to do so: the electrostatic potential for the transition structure within the catalyst (Figure 3d) appears uniform. The catalyst dipoles interact more strongly with the localised charge on the reactant (or product) ion-molecule complex than with the delocalised charges on the atoms of the transition structure.

However, when a pair of inert-gas atoms (grey spheres in Figure 3d) was placed on the N**^…^**C**^…^**N axis so as to impose repulsive interactions on both the reactant and transition structures sandwiched between them, the destabilising effect Δ*E*^R^_compress_ on the former could be adjusted (by appropriate choice of the fixed separation of the inert-gas atoms) to be significantly larger than the destabilising effect Δ*E*^T^_compress_ on the latter. The net effect of the attractive and repulsive components of the catalyst yielded Δ*E*^‡^_cat_ < Δ*E*^‡^_uncat_ (Figure 3e), because the preferential destabilisation of the reactant state by compression outweighed its preferential stabilisation by attractive interactions with the dipole array; alternatively, Δ*E*^R^_bind_ (= Δ*E*^R^_stabilise_ + Δ*E*^R^_compress_) < Δ*E*^T^_bind_ ( = Δ*E*^T^_stabilise_ + Δ*E*^T^_compress_) leading to net TS stabilisation.

**Scheme 2:**
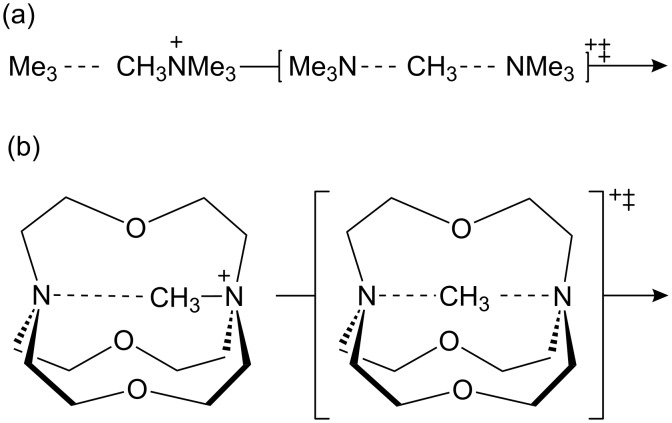
S_N_2 methyl transfer (a) uncatalysed and (b) within a cryptand cavity.

Later we proposed [16] a more realistic catalyst design for methyl transfer in the shape of inside-methylated [1.1.1]cryptand (Scheme 2). B3LYP/6-31G* calculations predicted the inter-bridgehead N**^…^**N distance in cryptand (b) to be 0.75 Å shorter than in the ion-molecule complex between trimethylamine and tetramethylammonium (a), indicating compression along the N**^…^**C**^…^**N axis, but more significantly the corresponding difference in the corresponding transition structures was only 0.35 Å. In other words, the change from reactant complex to transition structure was 0.4 Å less for the compressed reaction (b) than for the uncompressed reaction (a); moreover, the potential energy barrier for (b) was 22 kJ mol^−1^ less than for (a), and the α-D_3_ KIEs was more inverse (0.91 vs 0.93) for (b) than for (a). These results were consistent with the compression hypothesis for catalysis of methyl transfer.

### Origin of COMT catalytic power

To assess whether compression actually operates in COMT-catalysed methyl transfer, hybrid QM/MM calculations have been performed at the AM1/MM level [17–19]. The secondary α-D_3_ KIE for the COMT-catalysed reaction (Scheme 1) was calculated to be more inverse than for the same reaction in water [18], but this preliminary result was based upon single structures for the reactant complex and transition state of the enzymic and non-enzymic reactions. Recently we performed extensive AM1/OPLS/TIP3P simulations [19] with ensemble averaging to include the effect of thermal fluctuations in the enzyme and solvent environments to obtain a value for the α-D_3_ KIE = 0.82 ± 0.05, which is in excellent accord with the experimental value [11] of *V*^CH3^ /*V*^CD3^ = 0.83 ± 0.05 for methylation of 3,4-dihydroxyacetophenone with SAM at 37 °C catalysed by COMT. In contrast, we calculated *k*_CH3_ /*k*_CD3_ = 0.99 ± 0.16 for methylation of methoxide ion by *S*-methyldibenzothiophenium ion at 25 °C in methanol, as compared with the experimental value [12] of 0.97 ± 0.02. The computational results reproduce the experimental observation of a significantly more inverse value of α-D_3_ KIE for enzyme-catalysed than for uncatalysed methyl transfer in solution. However, the average values for the making and breaking bonds between C_α_ and, respectively, the nucleophile and nucleofuge in the nearly collinear TS for the COMT-catalysed reaction were computed as 2.06 ± 0.02 Å and 2.11 ± 0.01 Å, the sum of which is scarcely different from the sum of the corresponding average bond lengths, 2.18 ± 0.04 Å and 2.00 ± 0.04 Å, for the uncatalysed reaction. Thus the simulations did not provide any structural evidence for compression.

**Figure 4:**
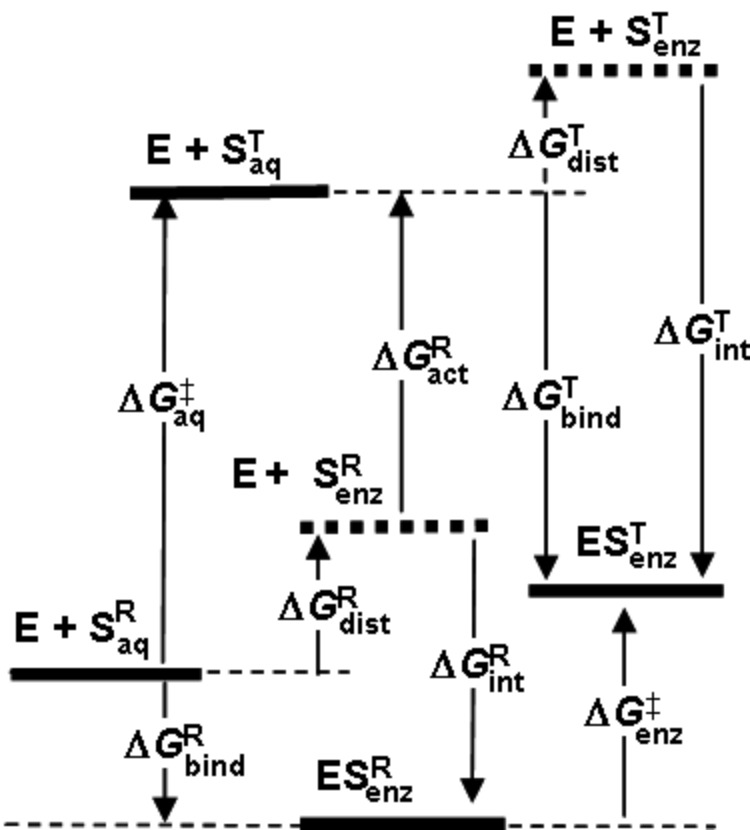
Free energy analysis of COMT catalysis.

It is instructive to analyse the various energetic contributions to catalysis (Figure 4) by COMT by means of appropriate computer simulations, as was done in an earlier study [17]. (N.B. The terminology and notation employed here differ from that work.) The potential of mean force (PMF), computed from MD simulations at the AM1/CHARMM/TIP3P level with umbrella sampling along a reaction coordinate defined as the difference in bond lengths from C_α_ to the nucleophile and nucleofuge, predicted a 44 kJ mol^−1^ increase Δ*G*^‡^_enz_ in free energy in going from the enzymic reactant complex ES^R^_enz_ to the enzymic transition state ES^T^_enz_ for the COMT-catalysed reaction at 300 K. An analogous PMF for exactly the same reaction occurring in water without COMT yielded a free energy minimum for a solvent-separated ion-pair reactant complex S^R^_aq_; if this species were taken as the reference state for both catalysed and uncatalysed reactions, the reduction in barrier height would simply be equal to Δ*G*^T^_bind_, the TS stabilisation. In the published analysis [17], the free energy barrier Δ*G*^‡^_aq_ = 82 kJ mol^−1^ for the uncatalysed reaction in aqueous solution was considered as the sum of two terms: (i) a distortion energy Δ*G*^R^_dist_ = 30 kJ mol^−1^ for going from S^R^_aq_ to a contact ion-pair S^R^_enz_ in solution having the same geometry as that of the substrate-derived part of the enzymic reactant complex ES^R^_enz_ and (ii) an activation free energy Δ*G*^R^_act_ = 52 kJ mol^−1^ to the transition state S^T^_aq_. The sum of Δ*G*^R^_dist_ and the interaction energy Δ*G*^R^_int_ is equal to the apparent binding energy Δ*G*^R^_bind_. The magnitude of the enzyme catalytic power Δ*G*^‡^_aq_ − Δ*G*^‡^_enz_ = 38 kJ mol^−1^ is equal to the difference in binding energies Δ*G*^T^_bind_ − Δ*G*^R^_bind_ of the enzyme with the TS and the solvent-separated ion-pair, neither of which was evaluated in the simulation. The difference Δ*G*^R^_act_ − Δ*G*^‡^_enz_ = 8 kJ mol^−1^ was considered to quantify the energetic influence of the environment – either protein or water – upon the substrate as it changes from the reactant state to the transition state. However, this analysis lacks consistency in one respect, because, although the structure of S^R^_enz_ is (by definition) geometrically the same for the substrate in both the enzyme active site and in aqueous solution, the structures of S^T^ in the two different environments are not the same. A fair point of criticism for the concept of TS binding in enzyme catalysis has been that the TS need not be the same for both the catalysed and uncatalysed reactions [8]. Consequently, the previous analysis [17] should be modified by recognising that the apparent binding energy Δ*G*^T^_bind_ is the sum of distortion energy Δ*G*^T^_dist_ and interaction energy Δ*G*^T^_int_.

The species S^R^_enz_ in water is well defined and amenable to computational investigation, although experimentally it is transient and may not necessarily correspond to a genuine intermediate. Similarly, species S^T^_enz_ in water is also well defined and amenable to computational investigation, although – unlike S^R^_enz_ in water – it was not considered in the previous work [17]. A fair evaluation of the energetic influence of the protein or water environment on the substrate as it changes from the reactant state to the transition state should be made by comparison of Δ*G*^‡^_enz_ with Δ*G*^R^_act_ + Δ*G*^T^_dist_, since in each case the structures are the same. Owing to the structural distortions of both the reactant and transition states in going from aqueous solution into the enzyme active site, the quantity Δ*G*^T^_bind_ − Δ*G*^R^_bind_ is an apparent catalytic power which differs from the intrinsic catalytic power Δ*G*^T^_int_ − Δ*G*^R^_int_ by virtue of the differential distortion energy Δ*G*^T^_dist_ − Δ*G*^R^_dist_.

### TS recognition in enzymic glycoside hydrolysis

**Scheme 3:**
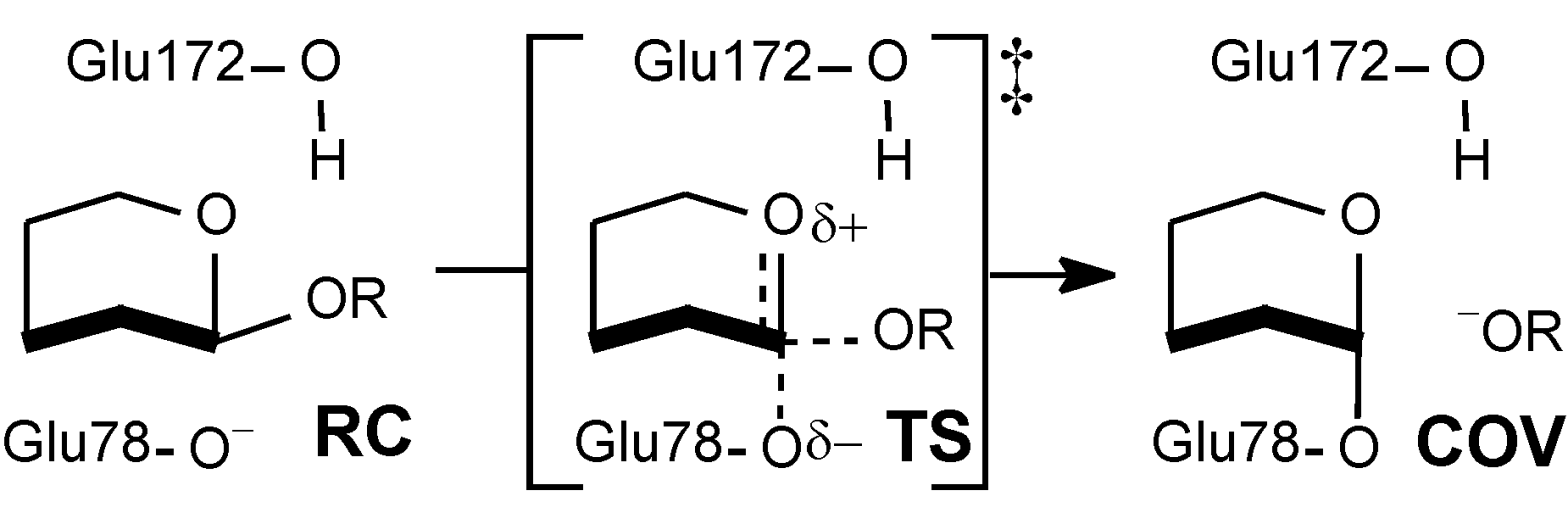
Formation of glycosyl-enzyme covalent intermediate COV.

The *endo*-1,4-β-xylanase (BCX) from *Bacillus circulans* catalyses the hydrolysis of xylan and β-xylobiosides with net retention of anomeric configuration by means of a double displacement mechanism involving a covalent glycosyl-enzyme intermediate. Formation and hydrolysis of this covalent intermediate occur via oxacarbenium ion-like TSs, with the assistance of two key active site glutamic acid residues [20]. Glu78 is deprotonated in the noncovalent enzyme-substrate reactant complex: it attacks the anomeric carbon of the substrate as a nucleophile and displaces the aglycone nucleofuge (Scheme 3). Glu172 is protonated in the reactant complex and plays a dual role of acid/base catalyst: in the glycosylation step it assists formation of the glycosyl-enzyme intermediate by donating a proton to the aglycone of the natural substrate, and in the subsequent deglycosylation step it serves as a base, deprotonating the attacking water molecule. Tyr69 donates a strong hydrogen bond to the nucleophilic oxygen atom (O_nuc_) of Glu78 in the reactant complex; in the covalent intermediate, this hydrogen bond is weaker, but a stronger interaction is formed between Tyr69 and the ring oxygen (O_ring_) of the proximal xylose moiety of the xylobioside substrate [21]. The phenolic oxygen (O_Y_) of Tyr69 is very important for catalysis, as evidenced by the observation that the Tyr69Phe mutant exhibits no detectable enzyme activity [22], and so it is an intriguing question to investigate the nature of this O_Y_H_Y_**^…^**O_ring_ interaction.

**Figure 5:**
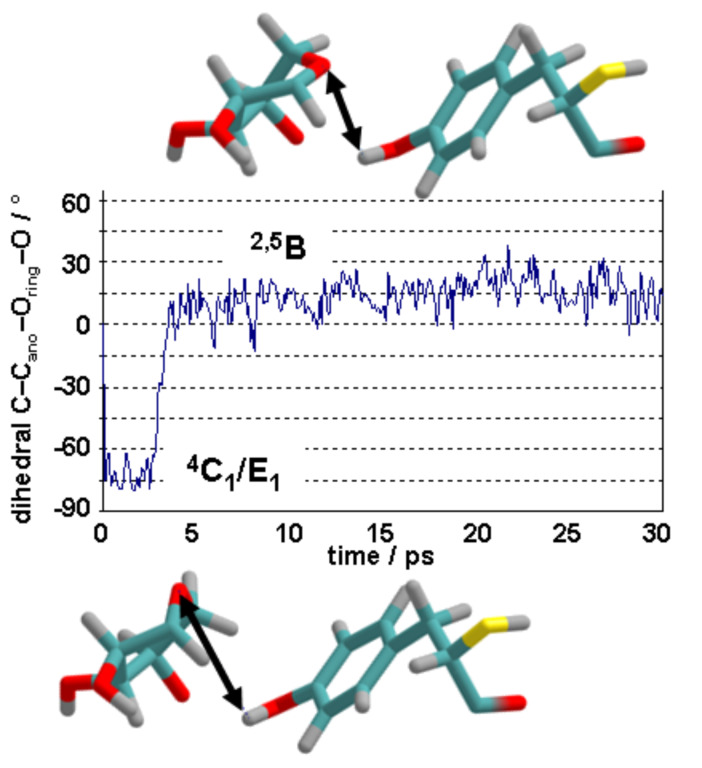
Conformational change of the xylose ring from chair (via envelope) with long O_Y_H_Y_^…^O_ring_ hydrogen bond to boat with short hydrogen bond, as shown by QM/MM MD simulation in active site of BCX.

MD simulations with the hybrid AM1/OPLS-AA/TIP3P method showed that both ^4^C_1_ chair and ^2,5^B boat conformers of phenyl β-xyloside remained stable in water during the course of 30 ps trajectories, even in the presence of propionate and propionic acid moieties to mimic Glu78 and Glu172 [23]. In contrast, analogous MD simulations for the ^4^C_1_ conformer of the reactant complex of phenyl β-xylobioside with BCX showed spontaneous transformation to the ^2,5^B conformer (Figure 5): the conformational change is accompanied by a marked decrease in the length of the O_Y_H_Y_^…^O_ring_ hydrogen bond. Moreover, analogous simulations for the Tyr69Phe mutant (lacking O_Y_) showed the chair to be stable, thereby confirming the key role of Tyr69 in preferentially stabilising the boat, with a relative free energy difference of about 20 kJ mol^−1^, by means of the O_Y_H_Y_**^…^**O_ring_ hydrogen bond [23].

**Figure 6:**
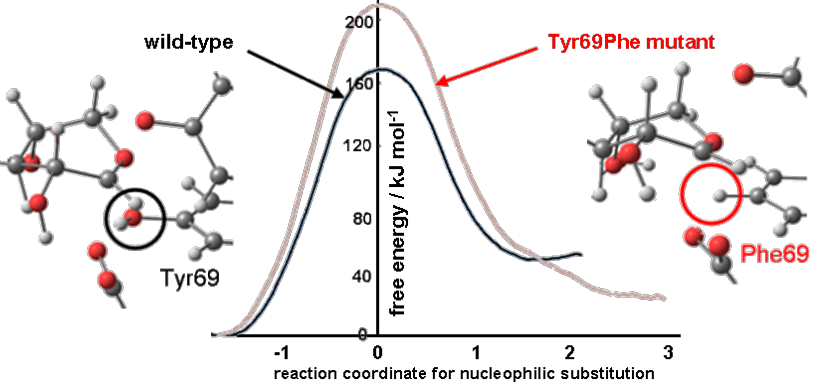
AM1/OPLS potentials of mean force for formation of glycosyl-enzyme covalent intermediate between 4-nitrophenylxylobioside and BCX wild-type (black) and Tyr69Phe mutant (red).

A two-dimensional PMF computed for 4-nitrophenyl β-xylobioside (the substrate employed in the experimental kinetics studies) with BCX using the same AM1/OPLS-AA hybrid potential, as a function of coordinates for nucleophilic substitution and proton transfer from Glu172, showed no requirement for protonation of the activated nucleofuge [24]. PMFs, with respect to the nucleophilic substitution reaction coordinate for both the wild-type and the Tyr69Phe mutant, computed with the same QM/MM MD method, revealed a decrease in free energy of activation of about 40 kJ mol^−1^ due to the presence of the single O_Y_ atom in BCX (Figure 6).

**Figure 7:**
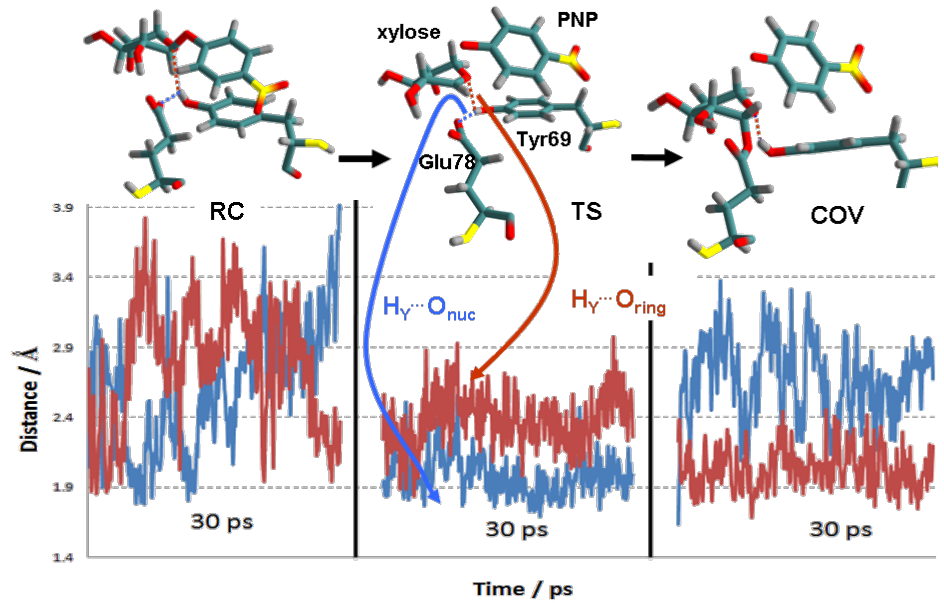
Hydrogen-bond distances H_Y_^…^O_ring_ (red) and H_Y_^…^O_nuc_ (blue) to boat conformer of RC, TS and glycosyl-enzyme COV intermediate, as shown by QM/MM MD simulation in active site of BCX.

Fluctuations in the hydrogen-bond distances H_Y_^…^O_ring_ (red) and H_Y_^…^O_nuc_ (blue) to the boat conformer of RC, TS and glycosyl-enzyme COV intermediate in the active site of BCX, as determined by 30 ps AM1/OPLS-AA MD trajectories, are shown Figure 7. Averaged over a longer (93 ps) trajectory for RC than shown here, the mean H_Y_^…^O_ring_ distance was significantly shorter (2.47 ± 0.49 Å) than H_Y_^…^O_nuc_ (3.29 ± 0.48 Å). On the other hand, H_Y_^…^O_nuc_ is consistently shorter (1.97 ± 0.14 Å) in the TS than H_Y_^…^O_ring_ (2.39 ± 0.20 Å), indicating that the hydrogen bond between Tyr69 and Glu78 is favoured, although both distances are shorter than in RC. In COV, however, H_Y_^…^O_nuc_ is once more longer than H_Y_^…^O_ring_, indicating that Tyr69 now donates its hydrogen bond exclusively to the xylose ring rather than to Glu78, although the average distance to the latter is similar to that in RC. Thus it appears that stabilisation of the TS is due to the transient presence of a shorter, stronger hydrogen bond to O_nuc_, which, of course, is absent in the TS for the Tyr69Phe mutant.

## Conclusion

Catalysts work by stabilising the TS relative to reactants, but the idea of designing a “catalyst” simply to bind strongly to the TS does not always work. Selective stabilisation of the TS for methyl transfer could be achieved in principle by means of compression, but in practice COMT catalyses by requiring less reorganisation of the electrostatic environment to go from RC to TS than is needed in aqueous solution, thereby achieving selective stabilisation of TS. The boat conformer of a xyloside substrate is favoured over the chair in the active site of BCX owing to a hydrogen bond from Tyr69 to O_ring_ of xylose, but preferential stabilisation of the TS in the wild-type relative to a Tyr69Phe mutant is achieved by means of a short, strong hydrogen bond from Tyr69 to the enzymic nucleophile. Catalysis is TS molecular recognition, and computational simulation may provide valuable insight into the causes of preferential stabilisation.
